# Supplementary material for: Wolbachia in Aedes koreicus: Rare Detections and Possible Implications
Source: Insects. 2022 Feb 21;13(2):216. doi: 10.3390/insects13020216 (PMC8879236; doi:10.3390/insects13020216)
Supplement: Supplementary file 1 [file insects-13-00216-s001.zip › insects-1589806.pdf]

SUPPLEMENTARY MATERIALS

Table S1. Mosquito, collected in field in 2019 and 2020, included in the study

Year collection	Province	Village	Latitude	Longitude	N°♂/N°♀
2019	Belluno	Belluno	46,140833	12,21555	20♂/19♀
	Vicenza	Maragnole Breganze	45,68552	11,605205	1♀
	Vicenza	Cogollo del Cengio	45,788363	11,420538	1♀
	Vicenza	Schio	45,711351	11,355359	1♀
	Vicenza	Zanè	45,723679	11,440547	2♀
	Vicenza	Sarcedo	45,695491	11,531565	1♀
	Vicenza	Calvene	45,765396	11,512042	1♀
	Udine	Premariacco	46,059641	13,387753	2♂
	Vicenza	Vicenza	45,557696	11,517407	1♀
	Padova	Padova	45,407717	11,873446	1♀
	Vicenza	Gallio	45,892927	11,546982	1♀
	Gorizia	Cormons	45,97019	13,46404	1♀
	Gorizia	Dolegna del Collio	46,042337	13,48574	1♀
	Padova	Baone	45,276222	11,697777	1♀
	Trieste	Duino-Aurisina	45,75631	13,65875	1♀
	Padova	Arcugnano	45,498066	11,550607	1♀
	Pordenone	San Giorgio della Rinchivelda	46,025438	12,821231	1♀
2020	Belluno	Feltre	46,017516	11,917255	3♀
	Padova	Baone	45,2549837	11,6862818	4♂/4♀
	Vicenza	Valli del Pasubio	45,763089	11,225205	5♂/1♀
	Vicenza	Carrè	45,746616	11,472657	2♂/3♀
	Vicenza	Laghi	45,825713	11,268992	4♂/5♀
	Vicenza	Lastebasse	45,915663	11,271447	5♀
	Verona	Monteforte d'Alpone	45,4449577	11,2852704	2♂
	Belluno	Belluno	46,12666	12,2334	4♂/4♀
	Belluno	Pedavena	46,051107	11,872342	5♂/5♀
	Pordenone	Montereale Valcellina	46,16652	12,660769	1♀
	Pordenone	Maniago	46,160859	12,679668	2♀
	Pordenone	Arba	46,143553	12,785998	4♂
	Pordenone	Fontanafredda	45,960318	12,54969	4♀
	Belluno	Sospirolo	46,142985	12,074161	5♂/3♀
	Padova	Cinto Euganeo	45,289394	11,702084	1♀
	Belluno	Limana	46,097651	12,179237	5♀
	Verona	San Zeno di Montagna	45,6442	10,7406	1♀
	Verona	Torri del Benaco	45,6079	10,7039	2♂
	Verona	Soave	45,4304848	11,2398901	1♂
	Treviso	Godega di Sant'Urbano	45,921427	12,418442	2♀
	Vicenza	Nanto	45,4294	11,5849	2♂
	Treviso	Domegge di Cadore	46.461897	12.419128	2♀
Total					148 (63♂/85♀)

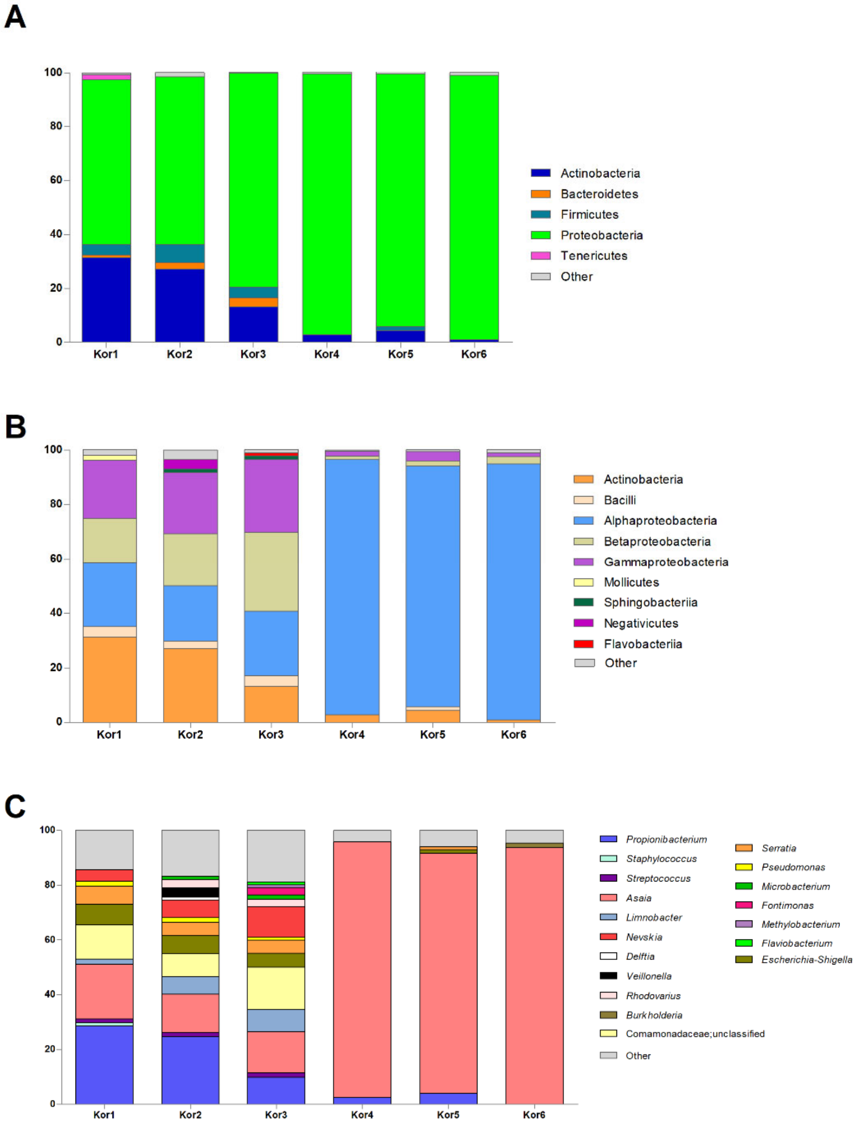

Figure S1. (A) Phylum level composition (B) Class level composition and (C) Genus composition (% of OTUs) in female *Ae. koreicus* mosquitoes. Only OTUs representing > 1% of the total reads are represented.
